# Draft genome sequence of *Penicillium citrinum* B9, a plant growth-promoting symbiont from barley rhizosphere

**DOI:** 10.1128/mra.00354-24

**Published:** 2024-07-05

**Authors:** Kenny J. X. Lau, Poonguzhali Selvaraj, Vayutha Muralishankar, Cheng-Yen Chen, Sahanna Muruganantham, Naweed I. Naqvi

**Affiliations:** 1 Temasek Life Sciences Laboratory, 1 Research Link, National University of Singapore, Singapore, Singapore; University of California Riverside, Riverside, California, USA

**Keywords:** fungus, mycobiont, penicillium, genome sequence, symbiosis

## Abstract

*Penicillium citrinum* strain B9 is a plant growth-promoting fungus isolated from Barley (Hordeum vulgare) rhizosphere. We report the first draft genome of *P. citrinum* B9 assembled using single-molecule real-time sequencing and Illumina reads. The assembled genome spans 31.3 Mb comprising nine contigs and 10,106 protein-encoding genes.

## ANNOUNCEMENT

*Penicillium citrinum*, a commonly occurring filamentous fungus of the family Aspergillaceae, has a worldwide distribution across all environments including soil, vegetation, and food products (1). Furthermore, *P. citrinum* also has been reported as an endophytic fungus that forms mutualistic relationship benefiting a broad range of plant genera as potential hosts (1, 2). *P. citrinum* isolate B9 is one such plant growth-promoting fungal strain initially isolated from the rhizosphere of barley. It has recently been shown to produce volatile organic compounds and bioactive phytohormones, such as gibberellins and auxin, which are involved in significant induction of growth in *Brassicaceae* hosts such as Choy Sum and Arabidopsis (3, 4).

Two-week-old barley plants grown in soil under greenhouse conditions were collected and processed under aseptic conditions. Surface sterilized roots (70% ethanol for 2 min followed by 10 washes in distilled water) were cut and homogenized using a sterile pestle and mortar, filtered, and plated on Prune juice agar medium. Individual fungal colonies were then purified and incubated in Complete Medium broth at 28°C for 7 days. Genomic DNA was extracted using the MasterPure Yeast DNA Purification kit (Lucigen, Biosearch Technologies) and used subsequently for PCR amplification. The isolate B9 was identified as *P. citrinum* based on the nucleotide sequence of the internal transcribed spacer (ITS) regions (ITS1 and ITS4) (3).

The whole-genome DNA library preparation, sequencing, and *de novo* assembly of the genome were performed by Macrogen (Macrogen, Singapore). Library preparation was carried out using the SMRTbell template prep kit, followed by single-molecule real-time (SMRT) sequencing on the PacBio RS II platform. Short reads were generated on the Illumina Novaseq platform (Illumina, USA) using whole-genome shotgun libraries. A total of 1,145,802 long reads were generated from the PacBio Sequel II platform (Pacific Biosciences, USA) which was used for *de novo* assembly with HGAP version 4.0 (4). It was then polished in Arrow (5) and error corrected using Pilon version 1.21 (6). The final assembly resulted in nine contigs with the largest contig that is 5,743,566 bp in length. A total of 19,916,750 short reads were generated from Illumina Novaseq platform (Illumina, USA). Reads with Phred score below 20 were filtered to obtain 13,329,018 reads. To validate the accuracy of the assembly, Illumina reads were used to map to the assembly. Of the filtered reads, 12,671,379 reads were mapped to the assembled contigs with a coverage of 99.99% and a depth of 58.65. A consensus sequence of the mapped reads was generated for taxonomic identification. Genome single-copy ortholog analysis was performed with BUSCO version 3 (7) using eukaryote_odb10 lineage data set, and it reported a genome completeness of 100.00%.

For genome annotations, MAKER v3.01.03 (8) was run with SNAP v2006-07-28 (9), and predictions were curated using InterProScan v5.30 (10) and Psi-Blast (11) against the eggNOG database v4.5 (12) with E-value of 1e-05. A total of 10,106 genes were predicted. The annotation results showed that the genome possesses mostly carbohydrate, amino acid, nucleotide, and lipid metabolism-related genes. Notably, it has 268 genes encoding for the biosynthesis of secondary metabolites and 450 gene functions for amino acid transport and metabolism. The genomic information could provide in-depth insight into *P. citrinum* genetics and biology, and comparative analyses in future studies on beneficial endophytic fungi and their plant growth-promoting secondary metabolites, such as gibberellins and cytokinin, for sustainable improvement of agricultural crop production.

## Data Availability

The complete genome sequence of *Penicillium citrinum* has been deposited in DDBJ/EMBL/GenBank under the accession number JAWVBB000000000 (https://www.ncbi.nlm.nih.gov/nuccore/2665898726). The version described here is JAWVBB000000000.1. The Bioproject number is PRJNA1033827, and Biosample number SAMN38047054 and the corresponding Sequence Read Archive (SRA) numbers are SRR26591480 (https://www.ncbi.nlm.nih.gov/sra/SRR26591480), SRR26591481 (https://www.ncbi.nlm.nih.gov/sra/SRR26591481), and SRR26591482 (https://www.ncbi.nlm.nih.gov/sra/SRR26591482).
